# Amoeba-Inspired Heuristic Search Dynamics for Exploring Chemical Reaction Paths

**DOI:** 10.1007/s11084-015-9450-5

**Published:** 2015-07-01

**Authors:** Masashi Aono, Masamitsu Wakabayashi

**Affiliations:** Earth-Life Science Institute, Tokyo Institute of Technology, 2-12-1 Ookayama, Meguro-ku, Tokyo, 152-8550 Japan; PRESTO, Japan Science and Technology Agency, 4-1-8 Honcho, Kawaguchi-shi, Saitama 332-0012 Japan; Department of Biomolecular Engineering, Tokyo Institute of Technology, 4259 Nagatsuta, Midori-ku, Yokohama, 226-8501 Japan

**Keywords:** Chemical reaction, Octet rule, Constraint satisfaction, Combinatorial optimization, Metastable states, Spatiotemporal dynamics

## Abstract

We propose a nature-inspired model for simulating chemical reactions in a computationally resource-saving manner. The model was developed by extending our previously proposed heuristic search algorithm, called “AmoebaSAT [Aono et al. 2013],” which was inspired by the spatiotemporal dynamics of a single-celled amoeboid organism that exhibits sophisticated computing capabilities in adapting to its environment efficiently [Zhu et al. 2013]. AmoebaSAT is used for solving an NP-complete combinatorial optimization problem [Garey and Johnson 1979], “the satisfiability problem,” and finds a constraint-satisfying solution at a speed that is dramatically faster than one of the conventionally known fastest stochastic local search methods [Iwama and Tamaki 2004] for a class of randomly generated problem instances [http://www.cs.ubc.ca/~hoos/5/benchm.html]. In cases where the problem has more than one solution, AmoebaSAT exhibits dynamic transition behavior among a variety of the solutions. Inheriting these features of AmoebaSAT, we formulate “AmoebaChem,” which explores a variety of metastable molecules in which several constraints determined by input atoms are satisfied and generates dynamic transition processes among the metastable molecules. AmoebaChem and its developed forms will be applied to the study of the origins of life, to discover reaction paths for which expected or unexpected organic compounds may be formed via unknown unstable intermediates and to estimate the likelihood of each of the discovered paths.

The satisfiability problem (SAT), one of the most studied constraint satisfaction problems, is stated as follows: Given a logical formula *f* involving *N* variables *x*_*i*_, does there exist an assignment *x*_*i*_ ∈ {1,0} (i.e., a combination of *N true*/*false* values) that satisfies *f*, which ensures that the overall formula *f* is *true*? For example, a problem instance $$ \begin{array}{llll}f\hfill & =\left(\neg {x}_1\vee {x}_3\vee \neg {x}_4\right)\hfill & \wedge \left({x}_1\vee \neg {x}_2\vee {x}_3\right)\hfill & \wedge \left({x}_1\vee {x}_2\vee {x}_3\right)\hfill \\ {}\hfill & \wedge \left({x}_1\vee {x}_2\vee {x}_4\right)\hfill & \wedge \left({x}_1\vee \neg {x}_3\vee \neg {x}_4\right)\hfill & \wedge \left(\neg {x}_2\vee {x}_3\vee {x}_4\right)\hfill \\ {}\hfill & \wedge \left({x}_2\vee \neg {x}_3\vee \neg {x}_4\right)\hfill & \wedge \left({x}_2\vee \neg {x}_3\vee \neg {x}_4\vee \right)\hfill & \wedge \left({x}_2\vee {x}_3\vee {x}_4\vee \right)\hfill \end{array} $$ has three solutions (*x*_1_, *x*_2_, *x*_3_, *x*_4_) = (1, 1, 1, 1), (1, 1, 1, 0), and (0, 1, 1, 0).

As the value of *N* increases, the total number of possible assignments grows exponentially as 2^*N*^ and no polynomial-time algorithm for finding a solution is known. SAT belongs to the particularly difficult class of problems known as NP (nondeterministic polynomial time). Moreover, SAT was the first problem shown to be NP-complete; this means that any problem in NP may be reduced to SAT in polynomial time [Garey and Johnson 1979]. For this reason, fast algorithms and systems capable of solving SAT may be applied to solve an extremely large number of problems. Many of these problems are closely related to applications that span a wide range of fields, including automatic inference, software/hardware verification, information security, and bioinformatics.

Aono et al. (2013) formulated the AmoebaSAT algorithm, which utilizes the spatiotemporal dynamics of a coupled system of 2*N* units corresponding to pseudopod-like branches of an amoeba, to solve the *N*-variable SAT problem. Each unit is assigned a variable name *i* ∈ {1, 2, ⋯, *N*} and a *true*/*false* value *v* ∈ {0, 1} and is associated with two variables *X*_*i*,*v*_ and R_*i*,*v*_. If, at discrete time step *t*, a resource is supplied to unit (*i*,*v*) (corresponding to the elongation of the amoeba branch), we denote this by ${R}_{i,v}(t)=1$, and we interpret this as meaning that the system is considering the assignment *x*_*i*_= *v.* If no resource is supplied, we write this as ${R}_{i,v}(t)=0.$

We define a variable *X*_i,*v*_ ∈ {−1, 0, 1} to represent the accumulated value of the resource-supply variable *R*_*i*,*v*_:

$$ {X}_{i,v}\left(t+1\right)=\left\{\begin{array}{ll}{X}_{i,v}(t)+1\hfill & \left(\mathrm{if}\;{R}_{i,v}(t)=1\;\mathrm{and}\;{X}_{i,v}(t)<1\right),\hfill \\ {}{X}_{i,v}(t)-1\hfill & \left(\mathrm{if}\;{R}_{i,v}(t)=0\;\mathrm{and}\;{X}_{i,v}(t)>-1\right),\hfill \\ {}\hfill & {X}_{i,v}(t)\left(\mathrm{otherwise}\right)\hfill \end{array}\right. $$

The quantity *X*_*i*,*v*_ may be understood as an abstract representation of the displacement from the equilibrium volume of the amoeba branches with one of the three values {−1, 0, 1}. In each step, the variables *X* = (*X*_1,0_, *X*_1,1_, *X*_2,0_, *X*_2,1_, ⋯ *X*_*N*,0_, *X*_*N*,1_) are transformed into the variable assignments $x=\left({x}_1,x{}_2,\cdots {x}_N\right)$ according to the following rule:

$$ {x}_i(t)=\left\{\begin{array}{ll}0\hfill & \left(\mathrm{if}\;{X}_{i,0}(t)=1\;\mathrm{and}\;{X}_{i,1}(t)\le 0\right),\hfill \\ {}1\hfill & \left(\mathrm{if}\;{X}_{x,1}(t)=1\;\mathrm{and}\;{X}_{i,0}(t)\le 0\right),\hfill \\ {}\hfill & {x}_i\left(t-1\right)\left(\mathrm{otherwise}\right)\hfill \end{array}\right. $$

We put *S*_*i*,*v*_(*t*) = 1 or *S*_*i*,*v*_(*t*) = 0 to indicate the application or non-application, respectively, of a signal (stimulus) that “bounces back” the supply of resources to ${R}_{i,v}$ (corresponding to an repulsive stimulus inhibiting the elongation of the amoeba branch). For now, we wish to focus on the leftmost clause (¬ *x*_1_ ∨ *x*_3_ ∨ ¬ *x*_4_) in *f*. If we have both *x*_1_ = 1 and *x*_3_ = 0, then we require that *x*_4_ should not be $1$ (i.e., *x*_4_ ≠ 1) in order for this clause to be *true*; indeed, otherwise we find (¬ (*x*_1_ = 1) ∨ *x*_3_ = 0 ∨ ¬ (*x*_4_ = 1)) = 0. For this reason, if at step *t* we have both *X*_1,1_(*t*) = 1 *and X*_3,0_(*t*) = 1, then at step *t* + 1 we apply a bounceback signal to *R*_4,1_(*t*) (i.e., we determine *S*_4,1_(*t* + 1) = 1, so that a resource is supplied to unit (4,1) with a certain low probability *P*_4,1_(*t* + 1). We call this rule a “bounceback rule”. Similarly, from the leftmost clause we can read off the bounceback rules *X*_1,1_(*t*) = 1 ∧ *X*_4,1_(*t*) = 1 ⇒ *S*_3,0_(*t* + 1) = 1 and *X*_3,0_(*t*) = 1 ∧ *X*_4,1_(*t*) = 1 ⇒ *S*_1,1_(*t* + 1) = 1. We proceed similarly to investigate all clauses in *f* to analyze mutual interdependencies between the variables and determine a set of all bounceback rules. On the other hand, for each unit (*i*,*v*) in which the bounceback signal is not applied (i.e., *S*_*i*,*v*_(*t*) = 0), the resource supply occurs (i.e., *R*_*i*,*v*_(*t*) = 1) with a certain high probability *P*_*i*,*v*_(*t*).

Under the bounceback rules defined in Aono et al. (2013), if a system state *X* = (*X*_1,0_, *X*_1,1_, *X*_2,0_, *X*_2,1_, ⋯ *X*_*N*,0_, *X*_*N*,1_) satisfies, for all (*i*,*v*), either the condition *X*_*i*,*v*_(*t*) = 1 ⇔ *S*_*i*,*v*_(*t*) = 0 or the condition *X*_*i*,*v*_(*t*) ≤ 0 ⇔ *S*_*i*,*v*_(*t*) = 1, then the system is “stable”. If this stability criterion is not satisfied, there is a high probability that the sign of *X*_*i*,*v*_(*t* + 1) differs from that of *X*_*i*,*v*_(*t*) depending on *S*_*i*,*v*_(*t*), and the system state *X* is unstable. Figure 1 shows an example of time evolution of AmoebaSAT.

**Fig. 1:**
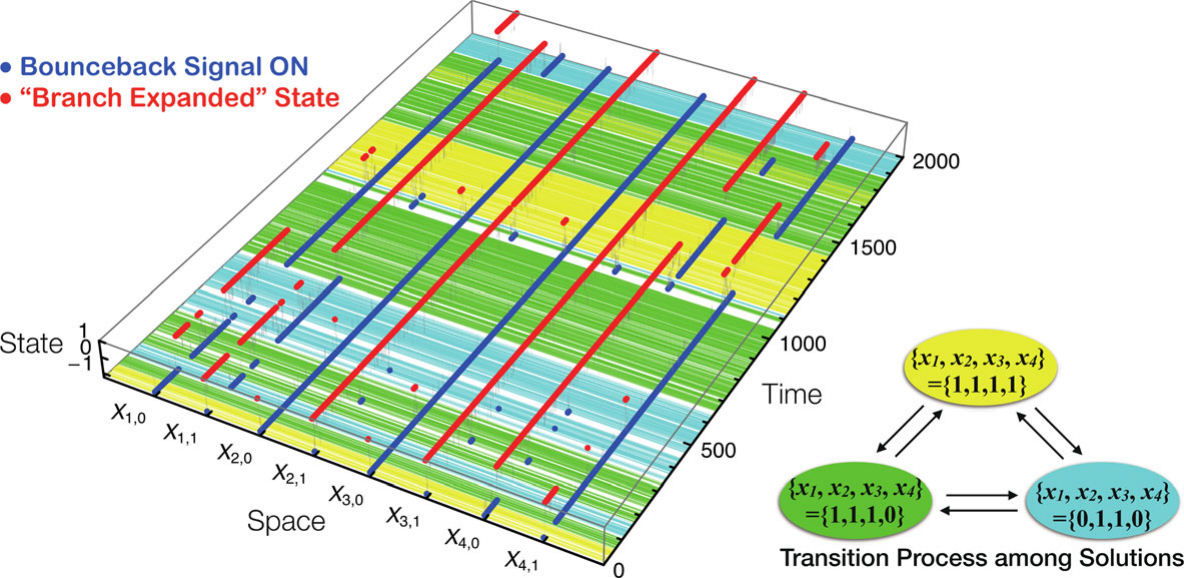
An example of time evolution of AmoebaSAT solving the problem instance *f*, which has three solutions

To evaluate the solution-searching performance of AmoebaSAT, we focused on a group of problems known as Uniform Random-3-SAT, in which all clauses are formed from three literals from the benchmark problems offered by the online SATLIB library [http://www.cs.ubc.ca/~hoos/5/benchm.html]. We selected 100 instances with *N* = 75 variables and 100 instances with *N* = 100 variables. For performance comparison, we considered WalkSAT, one of the categories of local search algorithms presently known to be the fastest heuristic methods for randomly generated 3-SAT instances [Iwama and Tamaki 2004]. WalkSAT configures its initial state by assigning all variables to random *true* or *false* values. Then the algorithm selects at random one clause from among the clauses that are not satisfied (i.e., are *false*) with the variable assignments at a given time and then chooses at random a single variable from within that clause to flip (changing 0 to 1 or 1 to 0). The algorithm then iterates this basic behavior. For each problem instance, we ran 500 Monte Carlo simulations of both the AmoebaSAT and WalkSAT algorithms and compared the average number of time steps (number of iterations) *t* required to arrive at the solution. We found that AmoebaSAT is able to find the solution with a speed orders of magnitude greater than that of WalkSAT [Aono et al. 2013].

Understanding the origins of the high performance exhibited by AmoebaSAT is a subject of current investigation. Whereas WalkSAT only updates one variable in each step, AmoebaSAT incorporates many processes, which collectively update multiple variables and evolve simultaneously while interfering with each other through the bounceback control mechanism. Analytical results have been obtained that suggest that this unique “concurrent search” feature of the algorithm is the source of its high performance.

As shown in Fig. 1, AmoebaSAT not only stabilized in a first-found solution but also exhibited probabilistic transition behavior among a number of solutions. The duration for which a solution is maintained, which corresponds to the time spent in one of metastable states, could be seen as representing with the concept of “thermodynamic stability”. The transition probabilities between two solutions vary depending on each pair of solutions, suggesting that the transition probability may contain information on “kinetics”. For example, the transition probability from a solution (*x*_1_, *x*_2_, *x*_3_, *x*_4_) = (1, 1, 1, 1) to (1, 1, 1, 0) is higher than that to (0, 1, 1, 0), because the former occurs with a bit flip whereas the latter requires two simultaneous bit flips that occur only infrequently.

Here we propose a new model, called “AmoebaChem,” that is an extended form of AmoebaSAT. In this article, we give only a brief explanation on AmoebaChem due to space limitations, and its detailed descriptions and results will be reported elsewhere. AmoebaChem considers a molecule as a (meta) stable solution in which several types of bounceback rules, which represent physical and chemical constraints including Lewis’s “octet rule,” are satisfied by all the atoms in the molecule. Figures 2 and 3 show the bounceback rules that are generated automatically when we input two nitrogen atoms and six hydrogen atoms. As shown in Fig. 4, each unit of AmoebaChem, which is depicted as a box marked with a 0 or 1 in Figs. 2 and 3, represents a bonding state of valence electrons owned by two atoms.

**Fig. 2:**
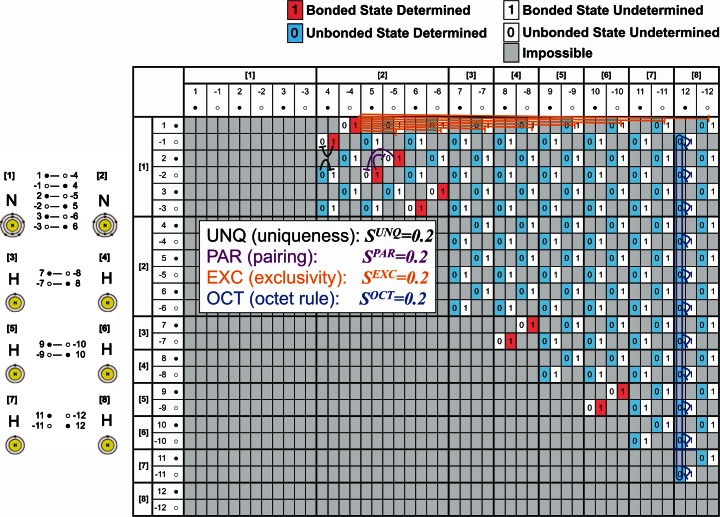
Bounceback rules in AmoebaChem, where two nitrogen atoms and six hydrogen atoms are introduced. The table shows that an N_2_ molecule and three H_2_ molecules are formed

**Fig. 3:**
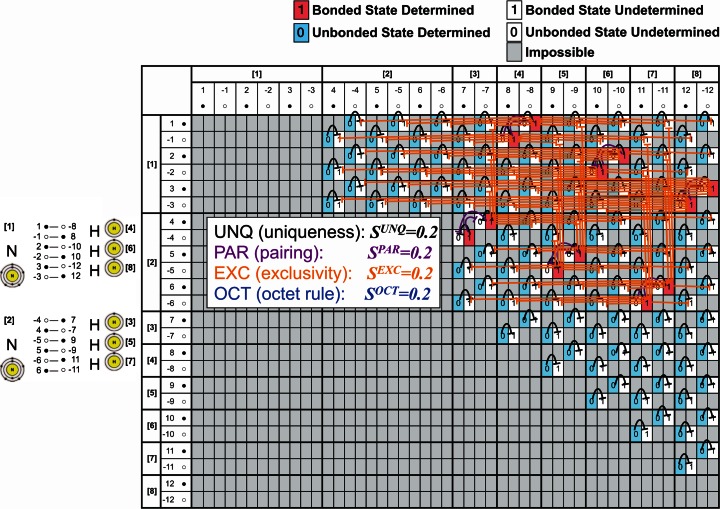
Bounceback rules in AmoebaChem, where two nitrogen atoms and six hydrogen atoms are introduced. The table shows that two ammonia molecules NH_3_ are formed

**Fig. 4:**
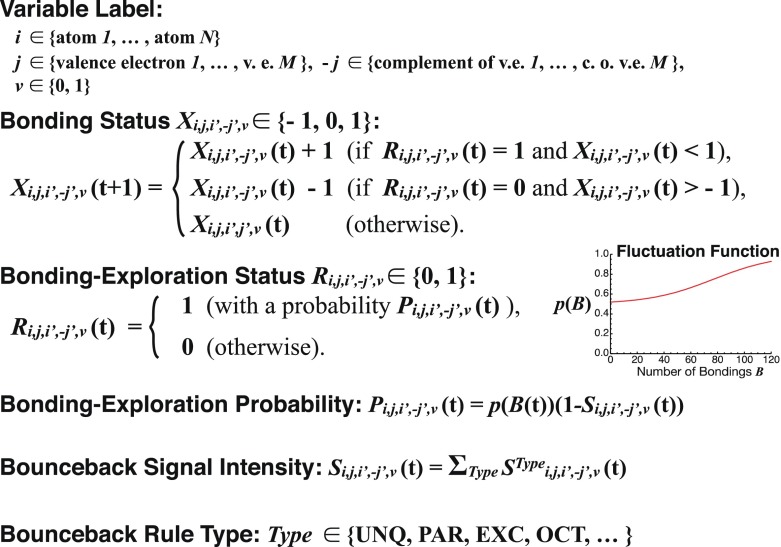
Formulation of AmoebaChem

A major difference between AmoebaChem and AmoebaSAT is that the bounceback signal intensities of the former (*S*^*Type*^(*t*)) are given as parameters taking continuous values in a real interval [0.0, 1.0] whereas that of the latter are binarized as *S*(*t*) ∈ {0, 1}. With smaller bounceback signal intensities, the behavior of AmoebaChem becomes more “unstable” since more aggressive probabilistic fluctuations (perturbations) are introduced, implying that the equivalent of “temperature” can be raised by lowering the parameters. Moreover, by changing the parameters, the equivalent of “kinetics” can be modulated. In fact, Fig. 5 shows that, for example, the transition probability from the initially given 1-N_2_-3-H_2_ state (Fig. 2) to a 2-NH_3_ state (Fig. 3) changed significantly as the parameter set was altered. It would be important to establish a methodology to tune the parameters for making the behavior of AmoebaSAT more realistic so that it can be consistent with experimentally observed reaction data.

**Fig. 5:**
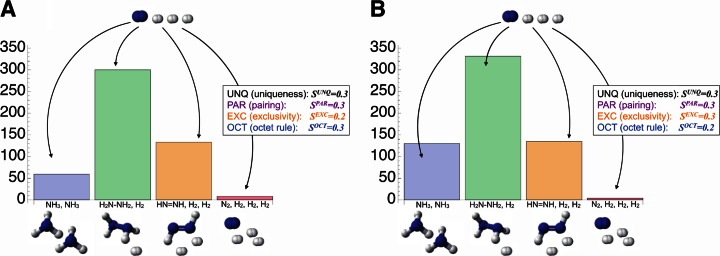
Distribution of first found stable states obtained after 500 trials of Monte Carlo simulation, where an N_2_ molecule and two H_2_ molecules are introduced initially. **a** and **b** show the results obtained with different parameter sets (bounceback signal intensities) as indicated in the insets

Although each unit of AmoebaChem introduced in this article represents a bonding state of valence electrons owned by two atoms, it can be replaced with other representations depending on purposes, for example, the number of bondings between two atoms. We can also simulate more realistic aspects in organic chemistry such as polarization, ionization, and radical reactions.

These models will be useful for finding unknown intermediate states that may exist before expected or unexpected organic products are formed.

If a variety of reaction paths can be found, we will be able to estimate the likelihood of each path by analyzing the durations and transition probabilities of the discovered intermediates.

We can also develop a simulator for RNA (secondary) structure prediction by considering each unit as representing a nucleotide and by introducing different bounceback rules that emulate complementary base-pairing rules and their related constraints. Furthermore, replacing nucleotides with amino acids, this simulator may be extended to protein (tertiary) structure prediction, if appropriate bounceback rules that represent physical and chemical constraints of protein folding such as hydrophobic-hydrophilic interactions can be introduced. Further investigations and developments will be devoted to advancing the study of the origins of life.
